# Human Bone Mesenchymal Stem Cell-Derived Exosomes Inhibit IL-1β-Induced Inflammation in Osteoarthritis Chondrocytes

**DOI:** 10.22074/cellj.2021.7127

**Published:** 2021-08-29

**Authors:** Liping Zhou, Haiwei Ye, Lizhen Liu, Yunhua Chen

**Affiliations:** 1 Chemical Pharmaceutical Research Institute, Taizhou Vocational and Technical College, Taizhou, Zhejiang, China; 2 Bone Marrow Transplantation Centre, First Affiliated Hospital of Zhejiang University School of Medicine, Hangzhou, Zhejiang, China

**Keywords:** Chondrocytes, Exosomes, Mesenchymal Stem Cells, Osteoarthritis

## Abstract

**Objective:**

Human bone marrow mesenchymal stem cell (hBMSC)-derived exosomes exhibit protective effects against inflammatory diseases. This study aimed to explore the effects of hBMSC-derived exosomes on osteoarthritis (OA) *in vitro* and its related mechanisms.

**Materials and Methods:**

In this experimental study, we characterised exosomes derived from hBMSCs by transmission electron microscopy, nanoparticle tracking and Western blot analysis. Cellular uptake of exosomes was observed by fluorescent microscopy. Cell viability of chondrocytes exposed to interleukin-1 beta (IL-1β) was determined by the Cell Counting Kit-8 (CCK-8). Real-time quantitative polymerase chain reaction (RT-qPCR) was used to determine expression levels of genes related to apoptosis, inflammation, cartilage collagen metabolism and mitogen-activated protein kinases.

**Results:**

Fluorescence microscopy revealed that hBMSC-derived exosomes could be taken up by chondrocytes. hBMSC-derived exosomes could significantly enhance cell viability of chondrocytes in response to IL-1β treatment. RT-qPCR showed significant up-regulation of Survivin, *Versican, IL-1β, IL-6, NF-κB, MMP-13, MAPK p38, JNK, ERK, Aggrecan* and *SOX9* expression levels by IL-1β treatment, while their mRNA expression levels decreased after co- culture with exosomes. The anti-inflammatory gene *TGF-β* was markedly suppressed by *IL-1β* treatment; however, we observed its expression after co-culture with exosomes. Additionally, the pro-inflammatory genes *IL-1β, IL-6, NF-κB, TNF-α* and *TNF-β* displayed significantly elevated expression levels in the IL-1β group and reduced expression levels after co-culture with exosomes.

**Conclusion:**

hBMSC-derived exosomes may play a protective role in chondrocytes through inhibiting cell apoptosis and the inflammatory response. These results will provide a novel therapeutic strategy for OA.

## Introduction

Osteoarthritis (OA) is a degenerative disorder of synovial joints of the hands, knees and hips that contributes to disability, reduced quality of life and mortality (1). OA remains a public health issue worldwide with an increasing incidence and is a major cause of disability, affecting about 240 million people globally, especially adults overthe age of 50 (2-4). Inflammation and inflammatory cytokines are considered to have a close association with the pathogenesis of OA, and studies have shown high expression levels of inflammatory mediators in OA patients (5, 6). Interluekin-1 beta (IL-1β), a pro-inflammatory mediator, promotes chondrocytes to release various proteolytic enzymes and other inflammatory cytokines, and has been reported to have a potential impact on the destruction of articular cartilage (7, 8). In addition, chondrocytes also play an essential role in the development of OA, and IL-1β-stimulated chondrocytes activate an inflammatory response that results in the leakage of many inflammatory mediators (9). Therefore, additional approaches that aim to suppress the effects of inflammatory mediators may provide a new therapeutic strategy for OA.

Currently, treatments for OA rely on a combination of pharmacological and non-pharmacological therapies to manage OA symptoms (10, 11). The standard drug treatments for OA are primarily non-steroidal anti-inflammatory drugs (NSAIDs), which relieve the symptoms of OA (12). However, long-term use of NSAIDs may lead to gastrointestinal and cardiovascular diseases (13). This underscores the need to discover new physiological and pharmacological pathways, which may be potential targets for novel agents for improved treatment of OA.

Mesenchymal stem cells (MSCs), with multidirectional differentiation potential and self-renewing capabilities, can be successfully differentiated into chondrocytes (14). Over the past two decades, MSCs, either alone or in combination with natural or synthetic scaffolds, have been used for cartilage repair, cellular therapy for OA and a range of related osteoarticular disorders (15, 16). The results of many studies show that the efficacy of MSC-based therapies is not only directly attributed to its differentiation function, but also its paracrine factors, especially exosomes (17-19). Exosomes are small, membrane-enclosed vesicles that can deliver cargo to recipient cells. MSC-derived exosomes function primarily as intercellular communication vehicles for the exchange of nucleic acids, bioactive lipids and proteins within cartilage and between joint tissues. It has been found that exosomes from human embryonic MSCs have a beneficial therapeutic effect on OA by balancing the synthesis and degradation of the chondrocyte extracellular matrix (20). Qi et al. (21) revealed that exosomes secreted by human MSCs inhibited mitochondrial-induced apoptosis of chondrocytes under inflammatory conditions via pathways that involved p38, ERK and Akt. However, the underlying mechanisms of MSC exosomes in alleviating OA are not completely understood.

Therefore, this study aimed to explore the role of exosomes isolated from human bone marrow MSCs (hBMSCs) in treating OA pathogenesis and its related mechanisms. These findings will improve our understanding of the occurrence and development of OA and may provide a novel strategy for the treatment of OA. 

## Materials and Methods

### Isolation and characterization of chondrocytes 

In this experimental study, chondrocytes purchased from Yunmi Biotechnology Company (Shanghai, China) were cultured in Dulbecco’s Modified Eagle’s Medium (DMEM, Gibco, Grand Island, NY, USA) supplemented with 10% foetal bovine serum (FBS, Gibco, Grand Island, NY, USA), 100 U/ml penicillin (Gibco, Grand Island, NY, USA) and 100 μg/ml streptomycin (Gibco, Grand Island, NY, USA). The chondrocytes were incubated at 37˚C under humid conditions and 5% CO_2_ . Once the chondrocytes reached 80-90% confluency, they were passaged.

An inverted microscope (Olympus IX70) was utilised to observe the morphology of the chondrocytes. The chondrocytes were identified by type II collagen immunohistochemistry staining. Briefly, chondrocytes were incubated overnight with anti-collagen II antibody (1:100; 15943-1-AP, Proteintech, Chicago, IL, USA). Thereafter, the chondrocytes were treated with horseradish peroxidase (HRP)-labelled secondary antibody (Jackson ImmunoResearch Laboratories, Inc., USA) at 37˚C for one hour. The colour was developed by diaminobenzidine (DAB, Beyotime Biotechnology, China), and the cells were counterstained with hematoxylin (Servicebio, China).

This research was performed after receiving the ethics approval from the Ethics Communication of First Affiliated Hospital of Zhejiang University (2018-IIT-34).

### Isolation and characterization of exosomes

### Isolation of exosomes

The hBMSCs used in this study were purchased from Cyagen Biosciences, Inc. (Santa Clara, CA, USA), and cultivated in DMEM supplemented with 10% FBS, 100 U/ml penicillin, and 100 μg/ml streptomycin. Exosomes in the cell supernatants were isolated by differential centrifugation. Culture supernatants of hBMSCs that contained the exosomes were harvested 48 hours after incubation with exosome serum-free containing medium by centrifugation at 500×g for 5 minutes, followed by centrifugation at 2000×g for 30 minutes at 4˚C. Afterwards, the cell culture supernatants were mixed with 16% polyethylene glycol 6000 (PEG-6000; Sangon, Shanghai, China) and incubated overnight at 4˚C. The cell culture supernatants were first centrifuged at 10 000×g for 60 minutes, and then ultra-centrifuged at 100 000×g for 70 minutes using an Optima-XE ultracentrifuge (Beckman Coulter, USA) to remove protein contaminants. Purified exosomes were suspended in phosphate buffer solution (PBS, Sinopharm Pharmaceutical Co. Ltd, China) and kept either at -80˚C for long-term preservation or at -20˚C for short term preservation. 

### Transmission electron microscopy

The hBMSCs-derived exosomes were washed three times in PBS for 5 minutes, and then fixed in 2% osmic acid (Aladdin Reagent, Shanghai, China) for 2 hours at 4˚C. Thereafter, the samples were dehydrated using increasing percentages of ethanol (Aladdin Reagent, Shanghai, China) as follows: 50% (15 minutes), 70% (15 minutes), 80% (15 minutes), 90% (15 minutes) and 100% (twice for 10 minutes). Finally, the exosome samples were embedded in Epon by progressively mixing Epon with acetone (Aladdin Reagent, Shanghai, China). Ultrathin sections were made and counterstained with uranyl acetate (Aladdin Reagent, Shanghai, China) and lead citrate (Aladdin Reagent, Shanghai, China). The ultrathin sections were examined using a JEM 1230 transmission electron microscope (JEOL, Japan).

### Nanoparticle tracking analysis

Nanoparticle tracking analysis (NTA) for exosomes was performed using NanoSight NS300 (Malvern Instruments Company, UK) to automatically track and determine the size of the exosomes in real time, as well as monitor and control their isolation and purification. The determination of the capture and analysis parameters were manually set and processed with NTA 2.2 Analytical Software Suite. 

### Western blot analysis

The protein concentrations of isolated exosome samples were measured by the bicinchoninic acid (BCA) method using a BCA Protein Assay kit (Boster Biological Technology Co., Ltd., China). Kit standards and samples were loaded into a 96-well plate and mixed with the working reagent. The plate was incubated for 30minutes at 37˚C and analysed with a spectrophotometer at 562nm (Eppendorf, BioPhotometer).

The protein samples were preheated at 100˚C for 5 minutes and the 20 µg samples were separated by polyacrylamide gel electrophoresis (SDS-PAGE) and transferred to polyvinylidene difluoride (PVDF) membranes. After blocking with 5% non-fat milk for 1 hour at 37˚C, the membranes were probed overnight with primary antibodies for CD9 (1:1000; Abcam, ab92726, USA), CD63 (1:1000; ABclonal, A5271, country) and CD81 (1:1000; Abcam, ab109201, USA) at 4˚C. After three washes in PBS-Tween20 (PBST) for 10 minutes, the membranes were incubated with the HRP-conjugated goat anti-rabbit IgG (1:1000; 111-035-003, Jackson ImmunoResearch Laboratories, Inc., USA) for 2 hours at 37˚C, washed again and incubated with chemiluminescent substrates. The blots were visualised using an enhanced chemiluminescence (ECL) system (Millipore, Bedford, MA, USA). 

### Cellular uptake of exosomes

Cellular uptake of exosomes was investigated by labelling with PKH67 (green fluorescent cell linker for general cell membrane labelling) using a commercial kit (PKH67GL-1KT, Sigma-Aldrich, USA) according to the manufacturer’s instructions. Briefly, the exosomes (700 μl) were diluted with Diluent C (1300 μl) followed by the addition of PKH67 dye (16 μl). Subsequently, the samples were mixed gently for 5 minutes before 1% bovine serum albumin (BSA, Sigma-Aldrich, USA) was added to bind the excess dye. The samples were then centrifuged at 120 000×g for 90 minutes to remove the supernatant. The samples were washed with PBS and resuspended for use.

The chondrocytes were seeded into 24-well plates and cultured overnight in serum-free medium. The next day, PKH67-labelled exosomes were added to each well and co-cultured with chondrocytes for 48 hours. After washing three times with PBS, the chondrocytes were fixed in 4% paraformaldehyde Biotechnology, China) at 26˚C for 10 minutes. After washing three times, the chondrocytes were stained with ActinRed (KeyGEN BioTECH, Nanjing, Jiangsu, China) in the dark at 26˚C for 30 minutes. After washing, the cell nuclei of the chondrocytes were stained with 4’, 6-diamidino-2-phenylindole (DAPI, Thermo Fisher Scientific Inc., USA) for 5 minutes in the dark. Thereafter, we observed cytoskeletal and nuclear alterations and the images were captured using a laser scanning confocal microscope (TCS SP8, Leica Microsystems Inc., USA).

### Effects of exosomes on the cell viability of IL-1β-induced chondrocytes

The chondrocytes were plated into 96-well plates at a density of 5×10^3^ /well and cultured overnight. The culture medium was then refreshed with DMEM that contained 10 ng/ml IL-1β for another 24 hours. Thereafter, the chondrocytes were treated with different concentrations of exosomes (0 μg/ml, 1 μg/ml, 2 μg/ml, 5 μg/ml and 10 μg/ml) for 24- and 48-hour periods. The cell viability of the chondrocytes was determined using a Cell Counting Kit-8 (CCK-8, Beyotime Biotechnology, China). We added 10 μl of CCK-8 reagent to each well and then incubated the plates for 2 hours. Absorbance was then measured at 450 nm using a microplate reader (Multiskan MK3, Thermo Fisher Scientific, USA). Each experiment was done in triplicate. Total RNA was extracted from the chondrocytes in the control group (chondrocytes cultured in DMEM), the IL-1β group (chondrocytes cultured in DMEM with 10 ng/ml IL-1β for 24 hours with subsequent exposure to 0 μg/ml exosomes for 48 hours) and the IL-1β+EXOs group (chondrocytes cultured in DMEM with 10 ng/ml IL-1β for 24 hours followed by exposure to 10 μg/ml exosomes for 48 hours) for real-time quantitative polymerase chain reaction (RT-qPCR) analysis.

### Real-time quantitative polymerase chain reaction

Total RNA was extracted using RNAiso Plus (TaKaRa, Japan) according to the manufacturer’s instructions. The isolated RNA was reverse transcribed into cDNA using the PrimeScript RT Master Mix (TaKaRa, Japan) in the PCR amplification instrument according to the manufacturer’s instructions. Table S1 (See Supplementary Online Information at www.celljournal.org) lists the primer sequences. The total reaction volume was 20 µl, and included 10 µl SYBR Premix EX Taq, 1 µl forward primer, 1 µl reverse primer and 8 µl sterilized distilled water that contained the cDNA. RT-qPCR was conducted using an ABI ViiA 7 Real-time instrument with the following reaction conditions: pre-denaturation at 95˚C for 2 minutes; 40 cycles at 95˚C for 15 seconds and 60˚C for 60 seconds; dissolution curve at 95˚C for 15 seconds, 60˚C for 60 seconds and 95˚C for 15 seconds. All values were normalized to the reference gene *GAPDH*, and relative gene expression levels were calculated using the 2^−ΔΔCt^ method. Data were obtained from three independent experiments performed in triplicate.

### Western blot of inflammatory factors

Total protein was extracted from the different groups using RIPA lysis buffer, and the protein concentrations were measured using a BCA Protein Assay kit following the manufacturer’s instructions. The protein samples (20 µg) were separated by SDS-PAGE and transferred to PVDF membranes. After blocking with 5% non-fat milk at 37˚C for one hour, the membranes were incubated at 4˚C overnight with primary antibodies for TGF-β (1:1000, Abcam, ab179695, USA), TNF-α (1:1000, Abcam, ab6671, USA), TNF-β (1:1000, Abcam, ab227929, USA), NF-kB (1:1000, Abcam, ab16502, USA), IL-1β (1:1000, Abcam, ab2105, USA), IL-6 (1:1000, Abcam, ab233706, USA) and GAPDH (1:1000, Proteintech, 60004-1-Ig, USA). After washing three times, the membranes were then incubated with goat anti-rabbit IgG (1: 1000) for 2 hours at 37˚C. The protein bands were visualized using the ECL system.

### Statistical analysis

All experiments were carried out at least three times, and data are presented as the mean ± standard deviation (SD). Statistical analyses were performed using SPSS software version 17.0 (SPSS Inc., Chicago, IL, USA). Comparisons between experimental and control data were evaluated by the student’s t test. P<0.05 was considered statistically significant.

## Results

### Morphology of chondrocytes

**Fig.1:**
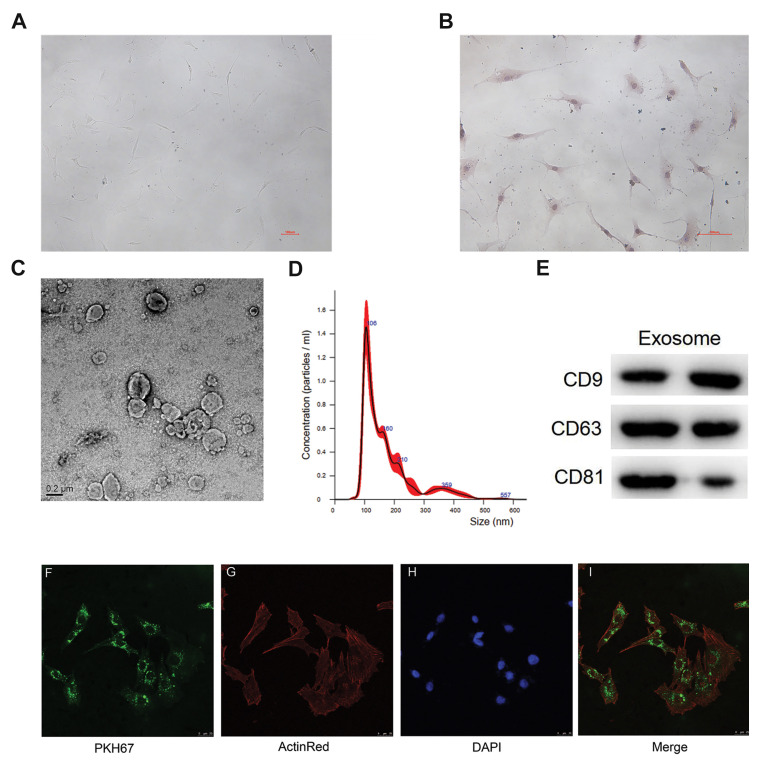
Identification of chondrocytes and exosomes isolated from human bone marrow stromal cells (hBMSCs) and exosome uptake by chondrocytes. **A.** Chondrocytes displaying spheroidal or elliptical morphology and contain a single nucleus (scale bar: 100 µm). **B.** Immunocytochemical staining of type II collagen (scale bar: 100 µm). **C.** Transmission electron microscopy images of hBMSCs-derived exosomes (scale bar: 0.2 µm). **D. **The size distribution of hBMSCs-derived exosomes by nanoparticle tracking analysis (NTA). **E. **Western blot analysis of exosome-specific CD9, CD63 and CD81 proteins. **F.** Exosomes were labelled with PKH67 (green fluorescent cell linker for general cell membrane labelling) (scale bar: 25 µm). **G.** PKH67-labelled exosomes were co-cultured with chondrocytes for 48 hours, and then chondrocytes were stained with ActinRed (red fluorescent) (scale bar: 25 µm). **H. **After co-culturing for a further 30 minutes, 4’, 6-diamidino-2-phenylindole (DAPI, blue fluorescent) was added (scale bar: 25 µm). **I.** Merged image of PKH67, ActinRed and DAPI. Most chondrocytes exhibited intracellular green fluorescence after incubation with exosomes (scale bar: 25 µm). PKH67-labelled exosomes were localized in the cytoplasm.

The morphology of chondrocytes under the light microscope (100×) were spheroidal or elliptical and contained a single nucleus (Fig .1A). The cell phenotype of the isolated chondrocytes was confirmed by type II collagen immunocytochemical staining, which showed that the cells stained dark yellow-brown. Therefore, positive type II collagen staining results indicated that the isolated cells were chondrocytes (Fig .1B).

### Characterisation of exosomes

Transmission electron microscopy analysis showed that exosomes isolated from hBMSCs were nearly round-shaped (Fig .1C). The size of the major particles was in the 110 nm range, which revealed that the nanovesicles were mainly exosomes (Fig .1D). In addition, Western blot analysis suggested that the exosomes expressed the characteristic surface markers CD9, CD63 and CD81, which are commonly used as surface markers for exosomes (Fig .1E).

### Exosome uptake by chondrocytes

Most chondrocytes exhibited intracellular fluorescence after incubation with exosomes, and the PKH67-labelled exosomes were localized in the cytoplasm (Fig .1F-I). This result indicated that exosomes could be taken up by chondrocytes through the plasma membrane after co-culture.

### Exosomes enhanced the cell viability of chondrocytes

**Fig.2:**
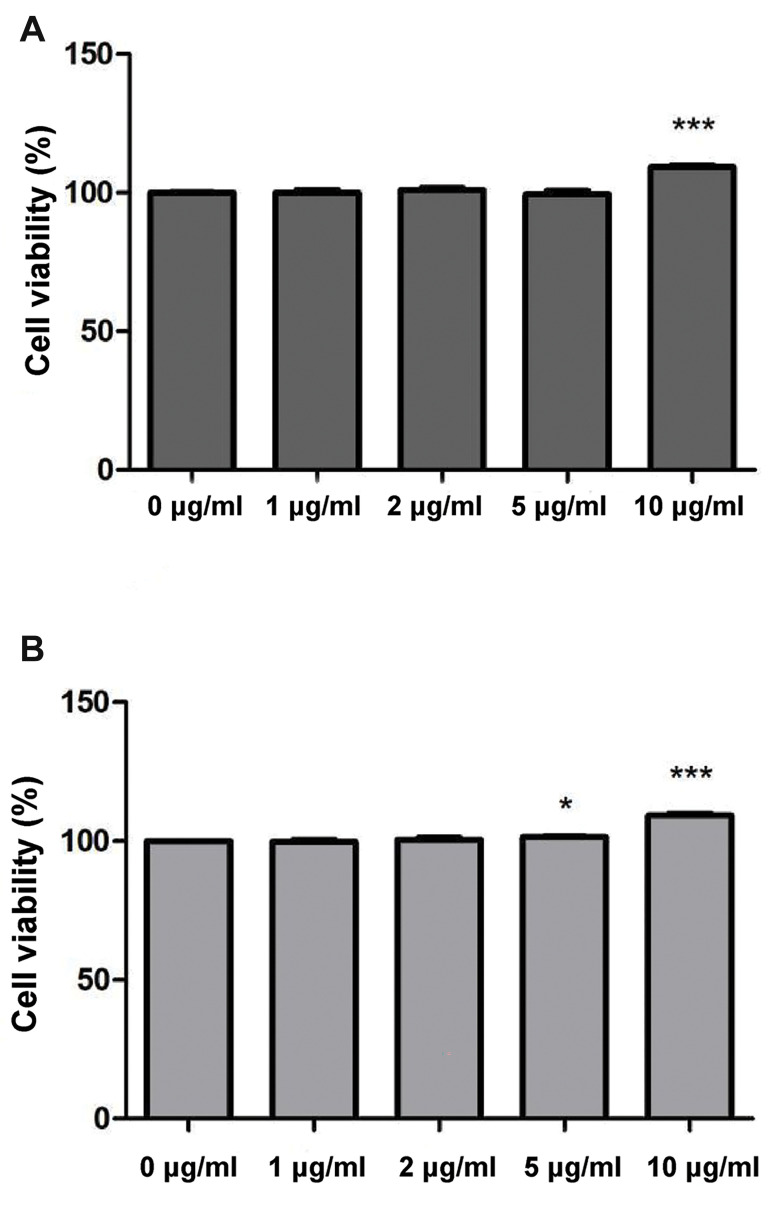
The cell viability of chondrocytes exposed to different concentrations of exosomes as determined by the Cell Counting Kit-8 (CCK-8) assay. **A.** Cell viability of chondrocytes exposed to different concentrations of exosomes for 24 hours. **B.** Cell viability of chondrocytes exposed to different concentrations of exosomes for 48 hours. *; P<0.05 compared with chondrocytes exposed to 0 μg/ml exosomes and ***; P<0.001 compared with chondrocytes exposed to 0 μg/ml exosomes.

The CCK-8 assay was utilized to evaluate the effects of exosomes on the cell viability of chondrocytes under inflammatory conditions. The results showed that exosomes at a concentration of 10 μg/ml significantly promoted cell viability after culturing for 24 hours (P<0.001, Fig .2A). Both the 5 μg/ml (P<0.05) and 10 μg/ml (P<0.001) exosomes significantly promoted cell viability after culturing for 48 hours (Fig .2B). Therefore, exosomes could enhance the cell viability of chondrocytes under* in vitro* inflammatory conditions.

### Effects of exosomes on the genes related to apoptosis and inflammation

**Fig.3:**
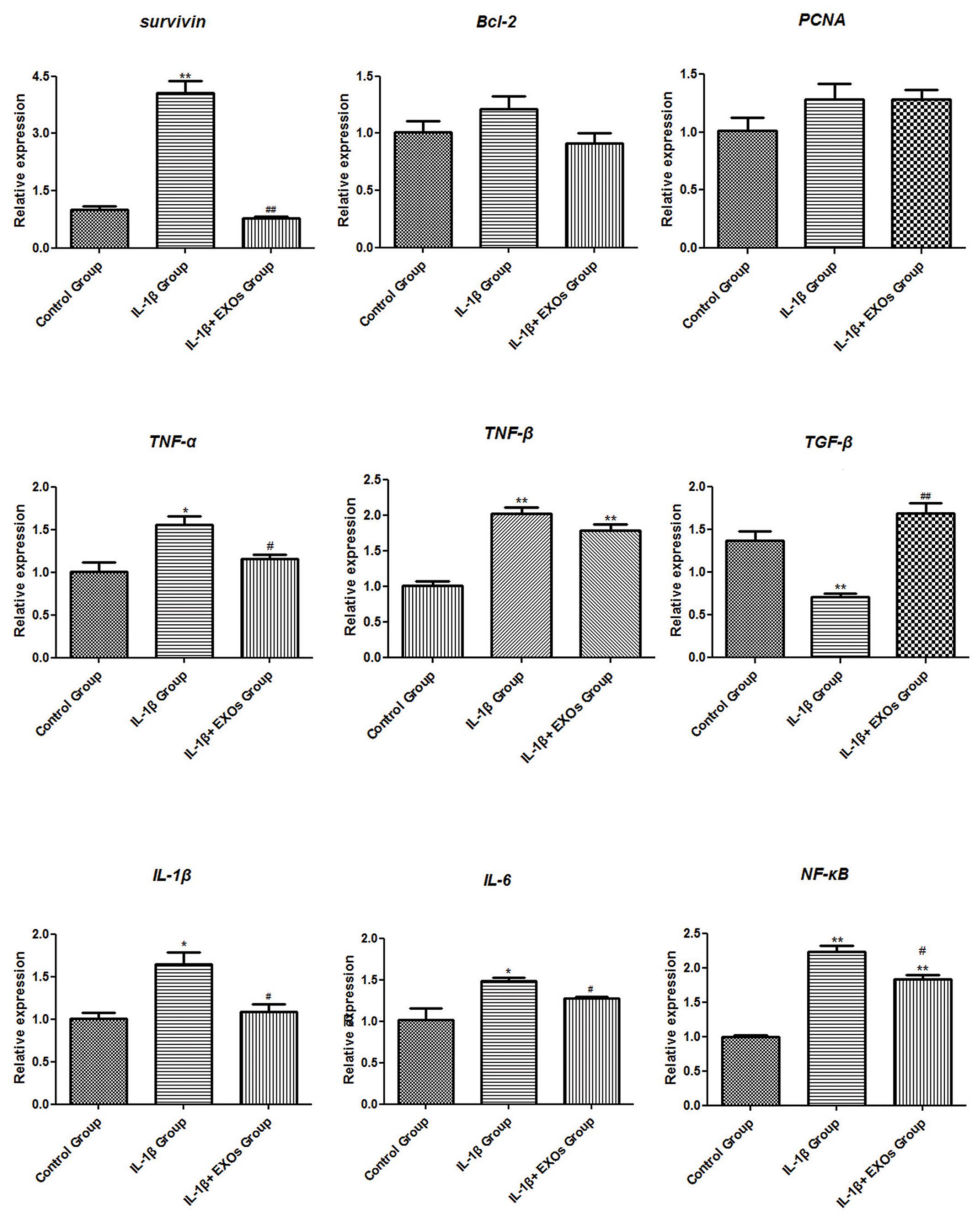
mRNA expression levels of genes related to apoptosis (*Survivin, BCL-2* and *PCNA*) and inflammation (*TNF-α, TNF-β, IL-1β, IL-6, TGF-β* and *NF-κB*) in chondrocytes were analysed by real-time quantitative polymerase chain reaction (RT-qPCR). Control group: Chondrocytes cultured in Dulbecco’s Modified Eagle’s Medium (DMEM), IL-1β group: chondrocytes cultured in DMEM with 10 ng/ml IL-1β for 24 hours followed by exposure to 0 μg/ml exosomes for 48 hours, IL-1β+EXOs group: chondrocytes were cultured in DMEM with 10 ng/ml IL-1β for 24 hours and then exposed to 10 μg/ml exosomes for 48 hours. TGF; Transforming growth factor, TNF; Tumor necrosis factor, NF-ΚB; Nuclear factor kappa-B, IL; Interleukin, EXOs; Exosomes, *; P<0.05 compared with the control group, **; P<0.01 compared with the control group, #; P<0.05 compared with the IL-1β group, and ##; P<0.01 compared with the IL-1β group.

RT-qPCR was conducted to detect the genes related to apoptosis, which included *Survivin, BCL-2* and *PCNA*. In the IL-1β group, mRNA expression for *Survivin* was significantly elevated compared with the control group (P<0.01). Exposure of the chondrocytes to 10 μg/ml exosomes for 48 hours (IL-1β+EXOs group) led to an obvious decrease in mRNA expression of *Survivin* (P<0.01). There was no statistically significant difference in the expression levels of *BCL-2* and *PCNA* in chondrocytes under the different treatments (Fig .3).

**Fig.4:**
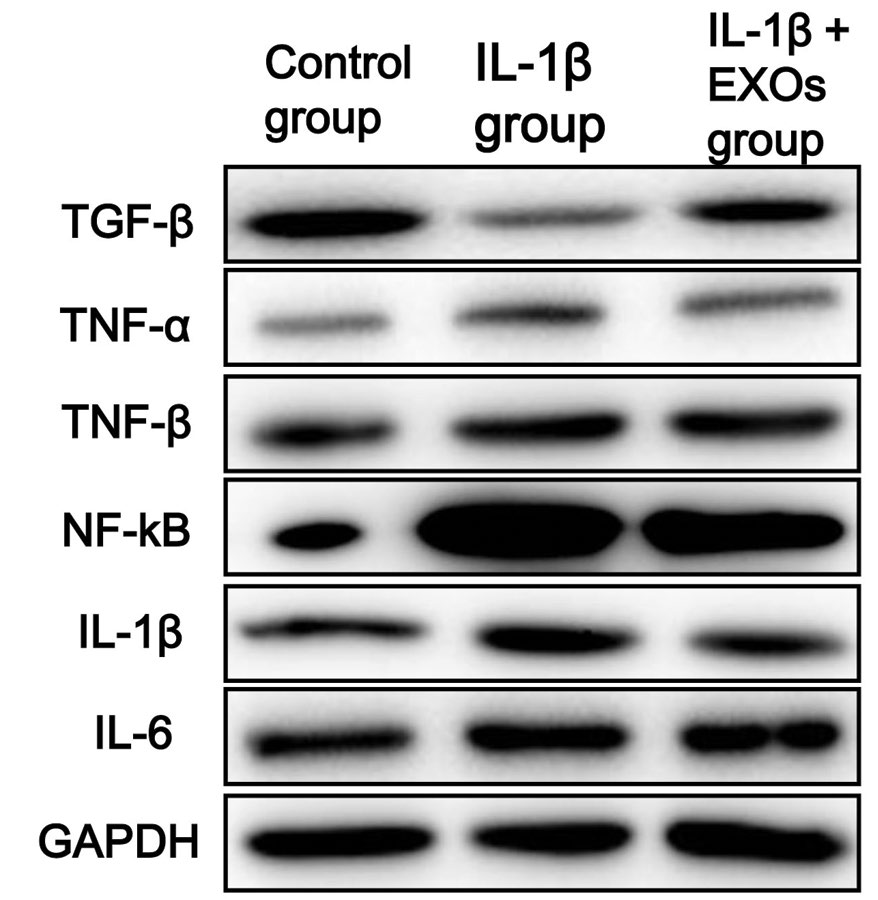
Protein expression levels of inflammatory factors (TGF-β, TNF-β, NF-KB, IL-1β, IL-6 and TNF-α) in chondrocytes as determined by Western blot analysis. Control group: chondrocytes cultured in Dulbecco’s Modified Eagle’s Medium (DMEM), IL-1β group: chondrocytes cultured in DMEM with 10 ng/ ml IL-1β for 24 hours followed by exposure to 0 μg/ml exosomes for 48 hours, IL-1β+EXOs group: chondrocytes cultured in DMEM with 10 ng/ml IL-1β for 24 hours and then exposed to 10 μg/ml exosomes for 48 hours. TGF-β; Transforming growth factor-β, TNF-β; Tumor necrosis factor-β, NF-ΚB; Nuclear factor kappa-B, IL-1β; Interleukin-1β, IL-6; Interleukin-6, TNF-α; Tumor necrosis factor-α, and EXOs; Exosomes.

The genes for the inflammatory factors of *TNF-α, TNF-β, TGF-β, IL-1β, IL-6 *and *NF-κB* were measured by RT-qPCR and Western blot analysis. RT-qPCR results showed that the anti-inflammatory gene *TGF-β *was significantly inhibited by 10 ng/ml IL-1β in the IL-1β group (P<0.05), while its level in the chondrocytes of the IL-1β+EXOs group increased after co-culture with exosomes (P<0.01). Chondrocytes from the IL-1β group showed marked elevations in the expression levels of the other pro-inflammatory genes *TNF-α, TNF-β, IL-1β, IL-6* and *NF-κB*, while their expression levels in chondrocytes of the IL-1β+EXOs group reduced after co-culture with exosomes (P<0.05, Fig .3). In addition, the changing trend of the inflammatory factors detected by Western blot analysis was consistent with the results obtained by RT-qPCR (Fig .4).

### Effects of exosomes on cartilage-specific markers

**Fig.5:**
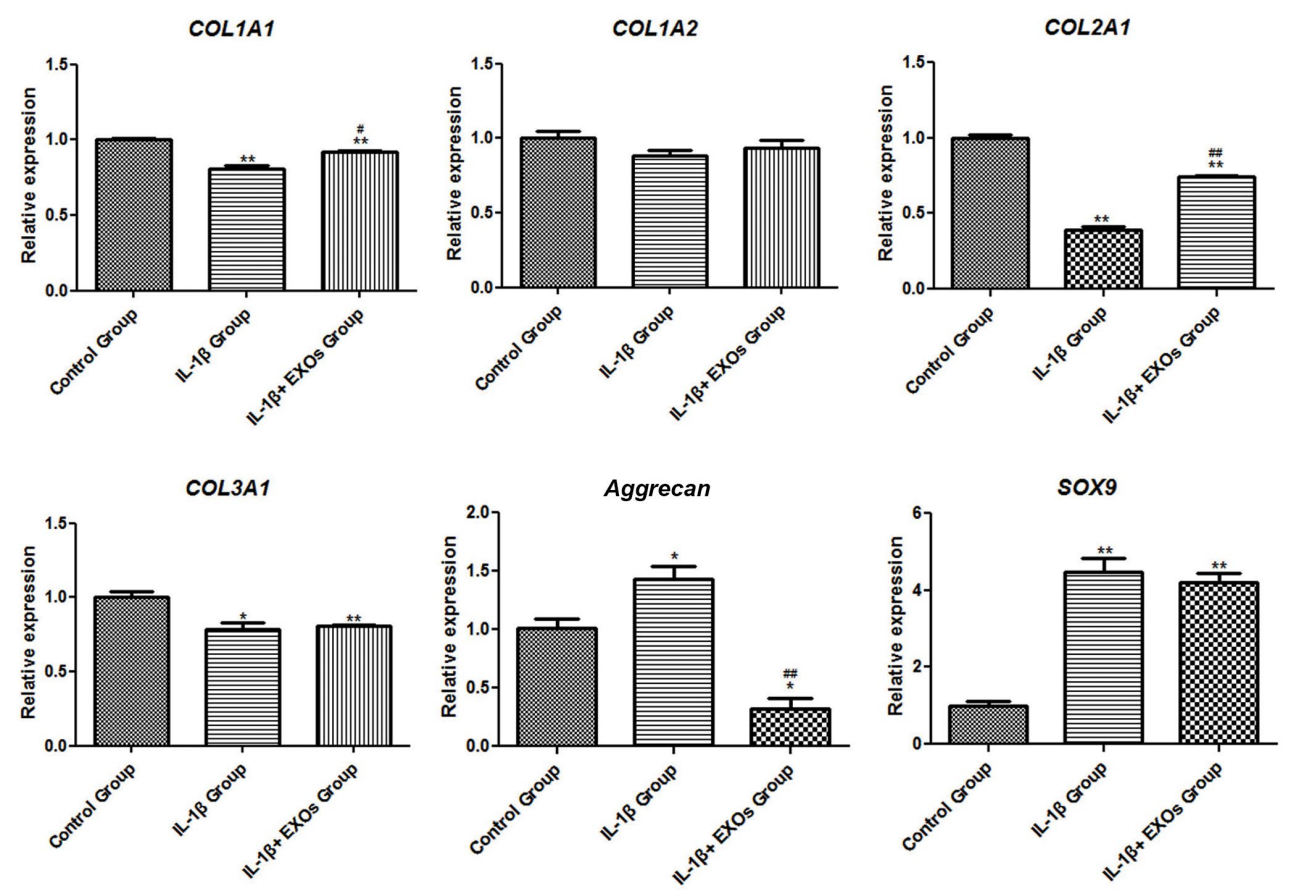
mRNA expression levels of the genes related to cartilage markers (*COL1A1, COL1A2, COL2A1, COL3A1, Aggrecan *and *SOX9*) in chondrocytes as analysed by real-time quantitative polymerase chain reaction (RT-qPCR). Control group: chondrocytes cultured in Dulbecco’s Modified Eagle’s Medium (DMEM), IL-1β group: chondrocytes cultured in DMEM with 10 ng/ml IL-1β for 24 hours followed by exposure to 0 μg/ml exosomes for 48 hours, IL-1β+EXOs group: chondrocytes cultured in DMEM with 10 ng/ml IL-1β for 24 hours and then exposed to 10 μg/ml exosomes for 48 hours. *; P<0.05 compared with the control group, **; P<0.01 compared with the control group, #; P<0.05 compared with the IL-1β group, ##; P<0.01 compared with the IL-1β group.

The expression levels of mRNA for cartilage-specific markers in chondrocytes, which included *COL1A1, COL1A2, COL2A1, COL3A1, Aggrecan* and *SOX9* were analysed by RT-qPCR. *COL1A1, COL2A1* and *COL3A1 *were down-regulated in chondrocytes from the IL-1β group (P<0.05), whereas they were up-regulated in the IL-1β + EXOs group (P<0.05, Fig .5). In contrast, we observed up-regulated expression levels of *Aggrecan* and *SOX9* in the IL-1β group and down-regulated expression levels of *Aggrecan* and *SOX9* in the IL-1β + EXOs group (P<0.05, Fig .5).

### Effects of exosomes on *CSPG4, MMP-13, Versican* and mitogen-activated protein kinases

**Fig.6:**
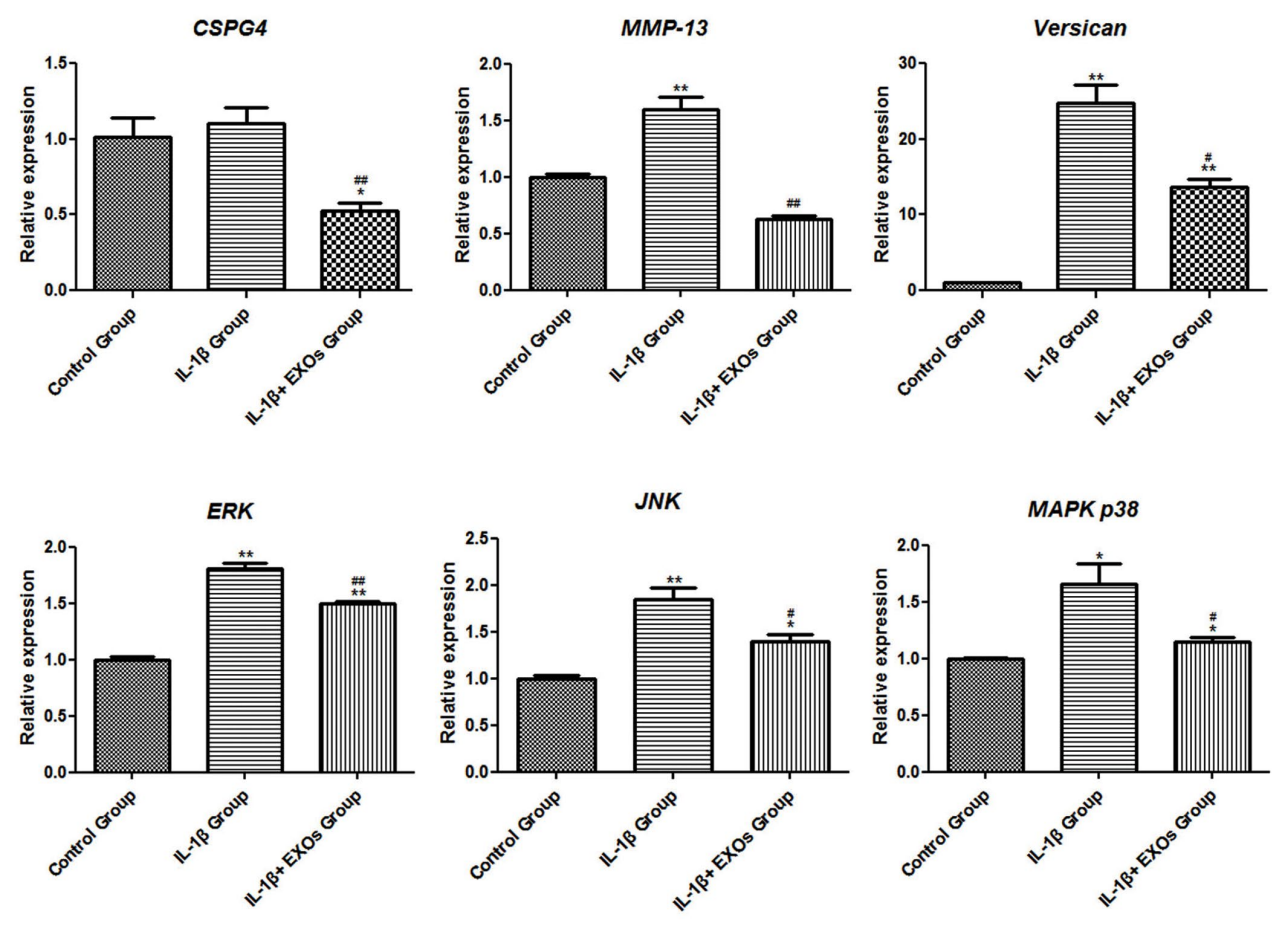
mRNA expression levels of *CSPG4, MMP-13, Versican, ERK, JNK *and* MAPK p38 *in chondrocytes as analysed by real-time quantitative polymerase chain reaction (RT-qPCR). Control group: chondrocytes cultured in Dulbecco’s Modified Eagle’s Medium (DMEM), IL-1β group; chondrocytes cultured in DMEM with 10 ng/ml IL-1β for 24 hours followed by exposure to 0 μg/ml exosomes for 48 hours, IL-1β+EXOs group: chondrocytes cultured in DMEM with 10 ng/ml IL-1β for 24 hours and then exposed to 10 μg/ml exosomes for 48 hours. *; P<0.05 compared with the control group, **; P<0.01 compared with the control group, #; P<0.05 compared with the IL-1β group, and ##; P<0.01 compared with the IL-1β group.

The mRNA expression levels of *CSPG4, MMP-13, Versican* and mitogen-activated protein kinases (*MAPK p38, JNK *and *ERK*) were measured. The results showed that the expression levels of *MMP-13, Versican, MAPK p38, JNK*, and *ERK* increased significantly after treatment with IL-1β (P<0.05). When exosomes were added to the chondrocytes and IL-1β, the expression levels of *CSPG4, MMP-13, Versican, MAPK p38, JNK* and *ERK* significantly down-regulated (P<0.05, Fig .6).

## Discussion

OA, which is characterized by the progressive destruction of articular cartilage, is the most common degenerative joint disease that leads to significant pain and disability in adults (22). Many obstacles still exist in the establishment of disease-modifying therapies for OA since the onset and development of OA involves complex molecular mechanisms related to synovial inflammation, the degeneration of articular cartilage and subchondral bone remodelling (23). In this study, we demonstrated that exosomes isolated from hBMSCs could significantly enhance the cell viability of chondrocytes in response to IL-1β treatment, which suggests that hBMSCs-derived exosomes exert a protective effect on chondrocytes under OA pathogenic conditions* in vitro*.

In previous studies, researchers observed high mRNA and protein expression levels of Survivin in rheumatoid arthritis and OA (24, 25). Survivin, which has a dual role of promoting cell proliferation and preventing apoptosis, is abundantly expressed in a majority of tumours and embryonic tissues; however, Survivin is not present in healthy differentiated cells (26). Baran et al. (27) have shown that high levels of Survivin are closely correlated with the destructive course of rheumatoid arthritis, and Survivin is an essential mediator of the interaction of arthritis with urokinase. Furthermore, *BCL-2*, a member of the apoptotic regulators, was found to be down-regulated in IL-1β induced cartilage degradation (28). *PCNA*, a key regulator of DNA replication, repair, cell cycle control, and apoptosis, has been reported to be up-regulated after berberine treatment of IL-1β-stimulated chondrocytes, which indicates an anti-apoptosis effect on OA chondrocytes (29). In our present study, RT-qPCR results revealed significant overexpression of *Survivin* under IL-1β treatment, and its mRNA expression clearly decreased after exposure to exosomes. However, there was no statistically significant difference in *BCL-2* and *PCNA* expressions of chondrocytes under the different treatments used in this study. Therefore, we speculate that hBMSCs-derived exosomes might exert a protective effect on chondrocytes by down-regulating *Survivin*. Further studies are required to explore the relationship between exosomes and the expression levels of *BCL-2* and *PCNA* in OA. 

It has been established that inflammation of the entire synovial joint (cartilage, subchondral bone and synovium) occurs during the development and progression of OA (30, 31). In this study, the anti-inflammatory gene *TGF-β* was markedly inhibited by IL-1β treatment, whereas higher expression levels were observed after co-culture with exosomes. The chondrocytes of the IL-1β group displayed a significant elevation in the expression levels of pro-inflammatory genes *TNF-α, TNF-β, IL-1β, IL-6* and *NF-κB*, while after co-culture with exosomes, their expression levels reduced in the chondrocytes. Conventional inflammatory factors, such as *IL-1β* and *TNF-α*, were reported to contribute to the systemic inflammation that leads to *NF-κB* activation in chondrocytes and synovial cells (32, 33). Gene expression profiling analysis of OA and control samples also revealed that inflammation signals contribute to OA pathogenesis through *MAPKs, NF-κB* activation and oxidative phosphorylation (34). *Versican* could influence the activation of inflammatory chemokines and further promote inflammation (35). *Versican* aggregation in the articular cartilage may play an important role in osteoarthritic cartilage (36). MMPs are a family of proteinases that contribute to the breakdown of the extracellular matrix and OA chondrocytes are characterized by elevated MMP-13 expression (37). RT-qPCR analysis showed up-regulation of *Versican, MMP-13, MAPK p38, JNK* and *ERK* after treatment with IL-1β, which is similar to what was observed in previous studies. These changes were attenuated significantly after the addition of exosomes to the chondrocytes. Thus, hBMSCs-derived exosomes may play protective roles in chondrocytes by reducing the inflammatory response through the inhibition of *Versican, MMP-13, MAPKs* and *NF-κB *activation.

We observed down-regulation of the cartilage-specific markers *COL1A1, COL2A1* and *COL3A1* in chondrocytes exposed to IL-1β and up-regulation of *Aggrecan* and *SOX9*. After exposure to exosomes, the expression levels of these five cartilage-specific markers displayed an opposite trend. *COL1A1, COL2A1* and *COL3A1* are genes that encode types I, II, and III collagen, which are specific to cartilage tissue and important for bone development, linear growth, structural framework and compression resistance in cartilage (38). It has been reported that aggrecanase degradation of type III collagen is associated with clinical knee pain in OA patients (39). Zhang et al. (40) indicated that *SOX9 *was up-regulated at the early stage of human OA and it participated in the progression of OA by mediating A disintegrin and metalloproteinase with thrombospondin motifs (ADAMTSs) induced cartilage degeneration. Thus, hBMSC-derived exosomes could suppress the expression levels of *Aggrecan* and *SOX9* in chondrocytes to relieve OA.

However, there are some limitations to our research. First, the effects of exosomes on OA by inhibition of the inflammatory response and mediation of the signalling pathways still require investigation. The protective effects of exosomes on apoptosis in chondrocytes also require further study.

## Conclusion

hBMSC-derived exosomes could exert a protective role in IL-1β-stimulated chondrocytes. The protective mechanisms may suppress the inflammatory response by inhibiting the expression levels of *TGF-α, IL-1β, IL-6, Versican, MMP-13, MAPKs* and *NF-kB*. Additionally, exosomes may control cell apoptosis by down-regulating *Survivin*. Exosomes may also have protective effects on OA by regulating the expressions of *COL1A1, COL2A1, COL3A1, Aggrecan* and *SOX9*. These findings improve our understanding of the occurrence and development of OA and provide a novel therapeutic strategy for OA.

## Supplementary PDF


