# Supplementary material for: Human Bone Mesenchymal Stem Cell-Derived Exosomes Inhibit IL-1β-Induced Inflammation in Osteoarthritis Chondrocytes
Source: Cell J. 2021 Aug 29;23(4):485–94. doi: 10.22074/cellj.2021.7127 (PMC8405079; doi:10.22074/cellj.2021.7127)
Supplement: Supplementary file 1 [file Cell-J-23-485-s01.pdf]

www.celljournal.org

Supplementary Information for

Human Bone Mesenchymal Stem Cell-Derived Exosomes Inhibit IL-1β-Induced Inflammation in Osteoarthritis Chondrocytes

Liping Zhou, M.M.[1]*, Haiwei Ye, M.D.[1], Lizhen Liu, M.D.[2], Yunhua Chen, M.B.B.S.[1]

1. Chemical Pharmaceutical Research Institute, Taizhou Vocational and Technical College, Taizhou, Zhejiang, China
2. Bone Marrow Transplantation Centre, First Affiliated Hospital of Zhejiang University School of Medicine, Hangzhou, Zhejiang, China

***Corresponding Address: Chemical Pharmaceutical Research Institute, Taizhou Vocational and Technical College, Taizhou, Zhejiang, China**
Email: lipingzhou788@aliyun.com*

Table S1: Primer sequences used in this study

Gene	Primer sequence (5´-3´)
Survivin	F: AGGACCACCGCATCTCTACAT<br>R: AAGTCTGGCTCGTTCTCAGTG
PCNA	F: GCGTGAACCTCACCAGTATGT<br>R: TCTTCGGCCCTTAGTGTAATGAT
TNF-β	F: ATGACACCACCTGAACGTCTC<br>R: CTCTCCAGAGCAGTGAGTTCT
IL-1β	F: ATGATGGCTTATTACAGTGGCAA<br>R: GTCGGAGATTCGTAGCTGGA
IL-6	F: CCTGAACCTTCCAAAGATGGC<br>R: TTCACCAGGCAAGTCTCCTCA
SOX9	F: AGCGAACGCACATCAAGAC<br>R: CTGTAGGCGATCTGTTGGGG
MMP-13	F: ACTGAGAGGCTCCGAGAAATG<br>R: GAACCCCGCATCTTGGCTT
Versican	F: GTAACCCATGCGCTACATAAAGT<br>R: GGCAAAGTAGGCATCGTTGAAA
TGFB1	F: GGCCAGATCCTGTCCAAGC<br>R: GTGGGTTTCCACCATTAGCAC
BCL-2	F: AGTACCTGAACCGGCACCT<br>R: CCACCAGGGCCAAACTGAGCA
COL2A1	F: TGCTGCCCAGATGGCTGGAGGA<br>R: TGCCTTGAAATCCTTGAGGCCC
Aggrecan	F: GTGCCTATCAGGACAAGGTCT<br>R: GATGCCTTTCACCACGACTTC
GAPDH	F: TGACAACTTTGGTATCGTGGAAGG<br>R: AGGCAGGGATGATGTTCTGGAGAG
